# Oxytocin receptors in the nucleus accumbens shell are necessary for the onset of maternal behavior

**DOI:** 10.3389/fnins.2024.1356448

**Published:** 2024-07-02

**Authors:** Shannah Witchey, Alexandra Haupt, Heather K. Caldwell

**Affiliations:** ^1^Laboratory of Neuroendocrinology and Behavior, Department of Biological Sciences, Kent State University, Kent, OH, United States; ^2^School of Biomedical Sciences and the Brain Health Research Institute, Kent State University, Kent, OH, United States

**Keywords:** adeno-associated virus, Cre recombinase, infanticide, knockouts, oxytocin receptor, pup abandonment, sensitized females, transgenic mice

## Abstract

In rodents, oxytocin (Oxt) contributes to the onset of maternal care by shifting the perception of pups from aversive to attractive. Both Oxt receptor knockout (Oxtr −/−) and forebrain-specific Oxtr knockout (FB/FB) dams abandon their first litters, likely due to a failure of the brain to ‘switch’ to a more maternal state. Whether this behavioral shift is neurochemically similar in virgin females, who can display maternal behaviors when repeatedly exposed to pups, or what neuroanatomical substrate is critical for the onset of maternal care remains unknown. To understand similarities and differences in Oxtr signaling in virgin pup-sensitized Oxtr FB/FB as opposed to post-parturient Oxtr −/− and Oxtr FB/FB dams, maternal behavior (pup-sensitized females only) and immediate early gene activation were assessed. Pup-sensitized Oxtr FB/FB females retrieved pups faster on day one of testing and had reduced c-Fos expression in the dorsal lateral septum as compared to virgin pup-sensitized Oxtr +/+ females. This differs from what was observed in post-parturient Oxtr −/− and Oxtr FB/FB dams, where increased c-Fos expression was observed in the nucleus accumbens (NAcc) shell. Based on these data, we then disrupted Oxtr signaling in the NAcc shell or the posterior paraventricular thalamus (pPVT) (control region) of female Oxtr floxed mice using a Cre recombinase expressing adeno-associated virus. Knockout of the Oxtr only in the NAcc shell prevented the onset of maternal care post-parturient females. Our data suggest that a pup-sensitized brain may differ from a post-parturient brain and that Oxtr signaling in the NAcc shell is critical to the onset of maternal behavior.

## Introduction

In mammals, the neuropeptide oxytocin (Oxt), known for its role in the facilitation of uterine contractions and milk ejection, acts within the central nervous to help initiate maternal behaviors (Burbach et al., 2001; Gimpl and Fahrenholz, 2001; Scott Young and Gainer, 2003). In the peripartum period there are marked increases in the peripheral release of Oxt as well as increases in Oxt within the paraventricular nucleus (PVN), the supraoptic nucleus (SON), and limbic regions of the brain (Zingg and Lefebvre, 1988; Kendrick et al., 1992). During late pregnancy and at the time of parturition there is a rapid upregulation, and subsequent down regulation, of the Oxt receptor (Oxtr), which is the single identified receptor subtype for Oxt. In rodents, Oxtr expression within numerous brain regions is upregulated by gestational day 22, maintained at parturition, and downregulated in the post-partum period (Broad et al., 1993; Pedersen et al., 1994; Meddle et al., 2007). Perhaps not surprisingly, the pattern of Oxtr upregulation in specific brain regions differs between parturient and pup-sensitized virgin rats within several brain regions, including the bed nucleus stria terminalis (BNST), the medial preoptic area (mPOA), and the medial amygdala (MeA) (Pedersen et al., 1994; Meddle et al., 2007). However, while parturient and pup-sensitized virgin females differ in their Oxtr expression, both have similar increases in peripheral Oxt concentrations (Higuchi et al., 1985). Thus, there are significant, and measurable, differences in Oxt neurochemistry in a mother who has given birth as compared to one that has not.

Data also suggest that Oxtr expression is precisely regulated around the time of birth. Specifically, temporally controlled Oxtr expression appears to be the greatest in brain regions that have been identified as part of the maternal behavior neural network (MBNN). The MBNN includes key neural sites where sensory cues and cortical inputs, as well as hormones and neurochemicals, coordinate and modulate the expression of maternal care (Bridges, 2016; Stolzenberg and Champagne, 2016). In the MBNN, the mPOA and BNST integrate pup-related sensory input and relay that information to the ventral tegmental area (VTA). The VTA then dampens nucleus accumbens (NAcc) output causing a disinhibition of the ventral pallidum (VP), which is permissive for the triggering of maternal responsiveness (Numan, 2006; Bridges, 2015). However, as previously mentioned, the way that pup-related sensory input is interpreted by the brain does differ in parturient versus virgin pup-sensitized females. Specifically, in mice, immediate early gene studies, e.g., c-Fos, suggest that there is differential neuronal activation within the BNST, the mPOA, the VTA, the NAcc core, and the NAcc shell in parturient versus virgin pup-sensitized females following exposure to pups (Matsushita et al., 2015).

Importantly, there is evidence that the Oxt system is important to maternal behavior in both virgin pup-sensitized and post-parturient females. Pharmacological studies have determined that Oxt acting via the Oxtr is particularly important for the onset of maternal behavior. In gonadal steroid-primed virgin female rats Oxt induces (Pedersen and Prange, 1979), and antagonism of the Oxtr impairs, the onset of maternal care, which suggests that Oxt plays a critical role in pup-sensitization (Fahrbach et al., 1985; Pedersen et al., 1985). Oxytocin neurons in the PVN have also been implicated in the social transmission of maternal behavior in virgin rats (Carcea et al., 2021). However, when looking specifically at the onset of maternal behavior, two brain regions have been identified as being critical, the mPOA and the VTA. The mPOA has estradiol-mediated increases in Oxtr expression that increase this regions sensitivity to pup stimuli (Pedersen et al., 1994). The VTA also expresses the Oxtr, specifically on dopaminergic and glutamatergic neurons (Peris et al., 2017), and it is here that Oxt signaling through its receptor results in increases in dopamine signaling in the NAcc, ultimately contributing to the onset of maternal care as well as individual differences in licking and grooming (Pedersen et al., 1994; Shahrokh et al., 2010). The timing of the Oxt signal is also important, as an Oxtr antagonist (Oxtr-A) administered at the time of parturition can block the onset of maternal behavior without having long-term effects on established maternal care (van Leengoed et al., 1987). This is consistent with evidence suggesting that once maternal behavior is established the administration of Oxt cannot enhance it further. Though, it is of note that administration of an Oxtr-A reduces pup-directed behaviors (Fahrbach et al., 1985; Pedersen and Boccia, 2003). Taken together these data suggest that Oxt signaling through the Oxtr is important in the initiation, and in specific instances the maintenance, of pup-directed maternal responses.

One way that the contributions of Oxt to maternal care have been interrogated is using mice with genetic disruptions of their Oxt system. Specifically, Oxt knockout (Oxt −/−) and Oxtr knockout (Oxtr −/−) mice. Interestingly, Oxt −/− and Oxtr −/− mice have no deficits in fertility, pregnancy, parturition, or maternal care, the latter in the instance that maternal care is initiated. Though, in both lines, due to their inability to milk eject, and thus nurse their offspring, there is complete litter loss within 24 h of parturition unless the pups are cross fostered (Nishimori et al., 1996; Takayanagi et al., 2005). That said, these mice do have issues with the initiation of maternal care. In a study from our lab, which separated nursing from maternal care, we found that *if* an Oxtr −/− dam initiated maternal care the quality of maternal care is not compromised. However, there is a robust pup abandonment phenotype, with 67% of Oxtr −/− dams abandoning their pups as compared to only 20% of Oxtr +/+ dams (Rich et al., 2014). A similar phenotype is observed in Oxtr forebrain conditional knockout (Oxtr FB/FB) dams, which can nurse their young. In these mice, 40% of dams abandon their first litter as compared to 10% in controls (Macbeth et al., 2010). Unfortunately, the data on pup-sensitization in Oxt −/− and Oxtr −/− virgin females are conflicting, with one study reporting deficits in retrieval latencies and pup licking in Oxt −/− females (Pedersen et al., 2006) and another reporting normal sensitization to pups in Oxtr −/− females (Takayanagi et al., 2005). While Oxtr −/− dams are known to spend equal amounts of time crouching over their pups as Oxtr +/+ dams in Oxtr −/− dam’s cages, pups are found to be scattered in the nest more frequently within the first 24 h (Takayanagi et al., 2005). These data highlight two things: (1) the importance of Oxtr signaling for the onset of maternal care and (2) how a pup-sensitized female’s Oxt system likely differs from that of a post-parturient female (Bosch, 2013; Kim et al., 2016; Kim and Strathearn, 2016; Numan and Young, 2016; Olazábal and Alsina-Llanes, 2016; Okabe et al., 2017; Yoshihara et al., 2018).

Pharmacological and genetic models have clearly demonstrated that Oxt is important for the onset of maternal care. Given the aforementioned pup abandonment phenotype observed in Oxtr −/− and Oxtr FB/FB dams (Macbeth et al., 2010; Rich et al., 2014), our laboratory has been focused on identifying the neural substrate(s) where Oxt acting via the Oxtr affects the onset of maternal care in post-parturient mice. We *hypothesized* that our observed pup abandonment phenotype was due to a failure of the brain to “switch” to a more maternal state. We predicted that Oxt acting via the Oxtr in brain regions important to the reward system, (e.g., VTA, NAcc, and VP) were necessary for a shift in the perception of pups from aversive stimuli to rewarding stimuli (Olazabal and Morrell, 2005).

To test this hypothesis, we examined neuronal activation by quantifying immediate early gene induction, i.e., c-Fos, in pup-sensitized Oxtr FB/FB females (Experiment 1) as well as in Oxtr −/− and Oxtr FB/FB dams one-hour following parturition (Experiment 2). Based on our findings from Experiment 2 we then went on to perform a targeted genetic disruption of the Oxtr to evaluate its contributions to the onset of maternal care (Experiment 3). These studies sought to identify unique and/or shared Oxtr-dependent activated brain regions in pup-sensitized females and post-parturient dams and use that information to determine where in the brain Oxtr signaling is required for facilitating the onset of maternal care.

## Materials and methods

### Animals

Adult females in this study were generated from three transgenic mouse lines: Oxtr −/−, Oxtr FB/FB, and Oxtr floxed (flox/flox) mice, along with their littermate controls. The creation of these independent mouse lines are detailed elsewhere (Lee et al., 2008; Macbeth et al., 2009). Oxtr −/− and Oxtr FB/FB mice were generated from heterozygous breeding pairs and Oxtr +/+ siblings used controls. The Oxtr flox/flox females were generated from homozygous lines. All mice were bred in the Kent State University vivarium and maintained on a 12:12 light/dark cycle, with food and water provided *ad libitum,* except during behavioral testing. All animals were weaned 21 days postpartum and housed in single-sex sibling groups. Tails were clipped at the time of weaning, DNA extracted and genotyping conducted as previously described (Lee et al., 2008). All experiments were conducted in accordance with the Kent State University Animal Care and Use Committee.

### Experiment 1: behavior and immediate early gene activation in virgin pup-sensitized Oxtr FB/FB females

#### Pup sensitization

Adult female Oxtr +/+ (*n* = 6) and Oxtr FB/FB (*n* = 8) mice were single housed one week prior to testing. Each female was exposed to four newly born C57BL/6 J pups (two males and two females) for 30 min for four consecutive days (postnatal day (PND) 1–4); we used this approach to avoid any confound associated with having litters of mixed genotype. Exposure started once the first pup was placed in the cage and only females who retrieved all four pups within the first five minutes continued to have their maternal behavior scored. If females failed to retrieve the pups within the first five minutes, all pups remained in the cage for the full 30 min to ensure sensitization. On PND4, one hour after the last exposure, females were euthanized by cervical dislocation, and their brains removed and placed into fixative (4% paraformaldehyde); in preparation for c-Fos immunocytochemistry.

#### Maternal behavior scoring

The behavior of pup-sensitized females was scored by an observer with no knowledge of genotype/treatment using Observer 5.0 (Noldus, Leesburg, VA). Pup-sensitized females were scored for (1) pup interactions (sniffing/licking); (2) nonsocial behaviors (resting alone/feeding); (3) nest building; and (4) self-grooming. To evaluate potential anxiety-like behaviors, nonsocial behaviors were further broken down into digging, rearing, and exploratory behaviors. Pup retrieval latencies were measured by determining the amount of time it took the female to retrieve the first and last pups. Maternal behaviors, except for pup retrievals, were analyzed within each day using a one-way ANOVA with genotype as the between factor. For this analysis, the amount of time the pup-sensitized females engaged in all behaviors (maternal and nonmaternal) were first summed, and the percentage of time the females engaged in each behavior was calculated. A *p* value of ≤0.05 was considered statistically significant. For pup retrievals, female had five minutes to retrieve the four pups, if they did not retrieve the pups then no maternal behavior was scored for that day and a 300 s latency score was assigned. The latency to retrieve the pups was analyzed across days and by genotype using a repeated measures ANOVA. If a *p* value of ≤0.05 was found, a Fisher’s least significant differences *post hoc* test was performed to determine which behaviors differed from one another.

#### C-Fos immunocytochemistry and quantification

Paraformaldehyde fixed brains were cut into three series of 50 μm free-floating sections using a Vibratome 1000Plus (Leica Microsystems, Buffalo Grove, IL) and stored at −20°C in cryoprotectant (0.1 M potassium phosphate buffer, sucrose, polyvinyl pyrolidione and ethylene glycol) prior to staining. At the time of staining, sections were washed six times for five min in 1XPBS, incubated in 1.5% hydrogen peroxide for five min, and then washed two times for five min. in 1XPBS. Following the washes, the sections were incubated in 1XPowerblock™ (Universal Blocking Reagent 10X, BioGenex, Fremont, CA) for 30 min. After blocking, sections were moved into the primary antibody (Santa Cruz Biochemicals, Santa Cruz, CA, USA, rabbit anti-c-Fos, sc-52) at 1:5000 in antisera diluent (PBS + 1% normal goat serum +0.3% Triton X-100) and incubated overnight at 4°C. The next day the sections were washed in 1XPBS three times for five min. and then incubated in avidin-biotin complex (Vectastain Elite ABC (Rabbit IgG), Vector Laboratories (Burlingame, CA, USA, PK-4001)) for one hour at room temperature. Sections were then washed in 1XPBS three times for five min. and incubated with diaminobenzidine (DAB) for 2–10 min. (Vector Laboratories, Burlingame, CA, USA, DAB substrate kit, SK-4100). To deactivate DAB, the sections were rinsed with 1XPBS followed by two washes in 1XPBS five min. The tissue was sequentially organized and mounted onto gel-subbed slides, allowed to air dry and cover slipped using DPX Mounting Medium (Sigma-Aldrich, DPX 06552).

c-Fos immunoreactive cells were quantified at 100X magnification on a Zeiss Axioscope by an observer with no knowledge of the experimental groups. iVision software (BioVision Technologies, Exton, PA) was used for image acquisition and processing. c-Fos-ir cells were manually counted within each neuroanatomical area. As described previously three sections per area, with sections being 100 μm apart, were bilaterally counted and averaged using set box sizes for each area [box sizes from Hasen and Gammie (2005)], which served to normalize count areas across animals. The areas measured included the dorsal and ventral aspects of the lateral septum, the LSD (732 × 754 pixels) and the LSV (380 × 338 pixels), respectively, the dorsal and ventral aspects of the BNST, the BNSTD (600 × 870 pixels) and the BNSTV (600 × 435 pixels), respectively, the mPOA (465 × 870 pixels), ventral palladium (200 × 250 pixels), MeA (990 × 870 pixels), periaqueductal grey (PAG) (300 × 550 pixels), lateral habenula brain region (LHB) (300 × 300 pixels), and NAcc core (500 × 500 pixels) and shell (500 × 500 pixels). These areas were identified based on the mouse brain atlas (Franklin and Paxinos, 2008). Comparisons between cell counts were made by area between genotypes using a one-way analysis of variance (ANOVA) (SPSS 16.0 for Mac, IBM, Armonk, NY). A result was considered statistically significant if *p* ≤ 0.05.

### Experiment 2: parturition-induced immediate early gene activation in Oxtr −/− and Oxtr FB/FB dams

#### Breeding

For the total Oxtr mouse line, Oxtr +/+ (*n* = 7) and Oxtr −/− (*n* = 9) and for the forebrain Oxtr mouse line, Oxtr +/+ (*n* = 8) and Oxtr FB/FB (*n* = 6) females were group housed 2–4 per cage for two weeks. To induce behavioral estrous, at the end of two weeks, male bedding was added to female cages (Whitten, 1956). The following day C57BL/6 J sires from our colony were placed into the female grouped-housed cages. Each day, for one week following pairing, females were checked each morning for the presence of a sperm plug to indicate a possible pregnancy. If a sperm plug was found, the male was removed, and the female was single housed. At the end of one week, all remaining males were removed from the females’ cages. Females that were single housed were then monitored for pregnancy based on weight gain. Any females, who were not pregnant, were paired again after two weeks had passed. Starting on gestational day 18, dams were checked hourly so that we could “catch” females about to give birth. Next, dams were euthanized by cervical dislocation one hour after the birth of the last pup, the completion of parturition was confirmed by gentle palpation of abdomen. Dam brains were collected and stored in 4% paraformaldehyde until processed for c-Fos immunocytochemistry as described for Experiment 1, with the addition of the PVN (300 × 175 pixels) and the VTA (275 × 200 pixels).

### Experiment 3: effects of targeted genetic knockdown of the Oxtr on maternal behavior

Based on the findings of Experiment 2, we evaluated whether disruption of Oxtr signaling in the NAcc shell (Experiment 3a) or the pPVT (control brain region) (Experiment 3b) resulted in any quantifiable changes in either the onset or expression of maternal behavior.

### Intracranial injections

#### Experiment 3a: nucleus accumbens

Prior to surgery, 16 Oxtr flox/flox sibling females were randomly assigned to two groups: (1) Cre recombinase adeno associated virus (Cre-IRES-GFP) (*n* = 8) or (2) control (IRES-GFP) (*n* = 8). The Cre-IRES-GFP (AAV2/2CMVCRE-wtIRESeGFP) and IRES-GFP (AAV2/2CMVeGFP), were purchased from the University of Iowa Viral Vector Core. At the time of surgery, animals were anesthetized using 2% isoflurane/oxygen mixture and placed into an Ultraprecise stereotaxic apparatus (David Kopf Instruments, Tujunga, CA). Once animals were secured in the ear bars, a midline incision was made across the top of the skull. The injection target was the NAcc shell and the coordinates used were, from bregma: anterior posterior +1.58, medial lateral ±0.7 and dorsal ventral −4.5 (from top of the skull). Burr holes were made using a hand-held drill (Dremel®, Racine, WI) with an engraving cutter bit (model #105, Dremel®, Racine, WI). Once the burr hole was created, the needle of a 2 μL Hamilton syringe (Hamilton Company, Reno, NV) was placed at the appropriate depth and allowed to sit for five minutes prior to injection to allow the brain to reposition. Bilateral injections of 0.5 μL of either the Cre-IRES-GFP or IRES-GFP were injected at a rate of 0.2 μL/min to limit damage. The needle remained in place an additional five minutes to allow for diffusion of the virus. The needle was then slowly removed, and the skin brought back together over the skull and closed with a wound clip. Following surgery, animals were administered 0.3 mL of warm saline (0.9%) intraperitoneally (i.p.) to aid in recovery and single housed with microisolator lids. Enrichment provided included a red dome and nestlets plus dams received peanuts at the time of cage changes. All animals were given two weeks to recover, to allow for maximal viral infection and the down regulation of Oxtr before being paired with a C57BL/6 J sire from our colony.

#### Experiment 3b: posterior paraventricular thalamus

To determine if the effects of Oxtr knockdown on the onset of maternal behavior were specific to the knockdown of the Oxtr in the NAcc shell, the pPVT was targeted as a control as it is known to express the Oxtr (Yoshida et al., 2009; Olazábal and Alsina-Llanes, 2016) and is important to maternal crouching behavior as well as the modulation of the stress response, and avoidance behaviors in the context of maternal care (Watarai et al., 2020; Kooiker et al., 2021). The same methods as indicated above for Experiment 3a were used to knockdown Oxtr in the pPVT, 12 Oxtr flox/flox sibling females that were randomly assigned to two groups: (1) Cre recombinase adeno associated virus (Cre-IRES-GFP) (*n* = 6) or (2) control (IRES-GFP) (*n* = 6). The injection coordinates used were from bregma: anterior posterior −1.46, medial lateral ±0.0 and dorsal ventral −3.0 (from top of the skull). The same post-surgery recovery was used as in Experiment 3a.

### Mating

Females that underwent stereotaxic surgery were paired with C57BL/6 J sires from our colony. All females were weighed weekly and once females appeared pregnant (or gained >3 g), males were removed from their cages. Two weeks after the male was removed females were re-paired if they did not appear pregnant. For Experiment 3a, if a female’s first litter did not survive, the dam was re-paired to evaluate the effects of the loss of the Oxtr within the NAcc shell on the care of subsequent litters.

### Pup observations

Dams were checked twice daily (900 h and 1,400 h), PND0 was designated as first day pups were present in the cage and completion of birth was confirmed via gentle palpation of dam’s abdominal section. A general health check of dams and pups occurred following parturition, including presence of a milk spot, number of pups (alive and dead), sex of pups and group pup weights. All litters were culled to 4 pups on PND0, 2 males and 2 females. To assure maternal behavior testing did not affect pups, pup weights and general health checks were recorded daily.

### Maternal behavior

The first measure of maternal behavior was the evaluation of pup abandonment. Any experimental animals that did not abandon their pups were then tested for maternal behavior from PND1 to PND3. For the measures of maternal behavior, the animal numbers were *n* = 8 for IRES-GFP and *n* = 2 for Cre-IRES-GFP for the NAcc shell and *n* = 4 for IRES-GFP and *n* = 5 for Cre-IRES-GFP for the pPVT. Prior to all behavioral testing, animals were acclimated to the testing space for one hour after lights out under dim red-light illumination. Pup retrieval was evaluated by first removing all pups from the home cage for five minutes while the dam’s behavior was videotaped. Pups were then scattered opposite to the location of the dam in the home cage and the five-minute pup retrieval task was videotaped. Pup retrievals were later quantified and included the latency to retrieve the first pup and the latency to retrieve all pups. If all pups were not retrieved within five minutes of being returned to the cage, a latency score of 300 s (s) as recorded and no maternal behaviors were scored. Following the five minutes of retrieval the dam’s behavior was videotaped for an additional 20 min. If all pups were retrieved, 20 min of maternal behavior was scored. Behaviors scored included, time on/off nest, licking/sniffing pups, nursing/crouching nest building, self-grooming, and rearing. Behavior was scored by an observer blind to genotype/treatment using Observer 5.0 (Noldus, Leesburg, VA) as previously noted and a repeated measures ANOVA was used to test for significant differences between groups.

### Postpartum behaviors

#### Elevated plus

On PND 4, experimental animals were tested on the elevated plus maze as.

previously described (Rich et al., 2014). Briefly, all animals were moved to the room one-hour prior to testing and the maze was illuminated at approximately 100 lux. Animals were placed in the center facing the closed arms of the elevated plus maze and electronically tracked for 10 min using EthoVision XT (Noldus, Leesburg, VA). The elevated plus maze consists of three quadrants: open arms (10 cm × 45 cm), closed arms (10 cm × 45 cm × 40 cm), and the center platform (10 cm × 10 cm). To assess anxiety-like behavior between treatment groups, the duration of time spent in the open and closed arms was summed and the percentage of time spent in the open and closed arms was determined.

#### Open field

On PND 5 experimental animals were tested in an open field test, as previously reported (Rich et al., 2014). Animals were moved to the testing room one hour prior to testing, the arena was illuminated at approximately 200 lux. All animals were tested in the open field made from Plexiglas measuring 45.5 × 45.5 × 30 cm. Animals were placed into the center of the open field and movement tracked for a total of 20-min by Ethovision XT (Noldus, Leesburg, VA). For analysis of anxiety-like behavior, the field was separated into two parts, an inner arena (measuring 32 × 32 cm) and outer arena. The tracking system quantified the amount of time spent in the inner versus outer arena. From this, the percentage of time spent in the inner and outer arenas was calculated.

#### Forced swim

The forced swim test was administered on last day of testing (PND 6) to assess depressive-like behaviors. Animals were moved to the testing room one-hour prior to testing, which occurred during the dark phase. Animals were placed into a 19 cm diameter cylindrical tank which was ¾ full of room temperature (~21°C) water. Animals were videotaped for six minutes and for the duration of the test they were observed for any signs of distress. All dams were then returned to a clean cage to which their nest and pups had been moved. Forced swim videos were later scored using Observer XT 9 (Noldus, Leesburg, VA) for swimming or floating for a total of four minutes, beginning at minute two, to allow for acclimation. To reduce observer error associated with transition, we used a sampling method of scoring. Behavior was scored every five seconds as either swimming behavior, two or more paws moving to propel the mouse, or float behavior consisting of no paws moving or two or fewer paws moving slightly only to stabilize the mouse in the water. The number of float and swim behaviors scored were summed and the percentage of swim and float behaviors scored was determined. All postpartum behavioral measurements were compared between genotypes using a one-way ANOVA with treatment groups as the main factor. A *p*-value of ≤0.05 was considered statistically significant.

### Site checks

Following behavioral testing, animals were euthanized, brains fast frozen on dry ice and stored at −80°C for injection site confirmation using green fluorescent protein (GFP) immunostaining. Tissue was sectioned at 12 μm in a − 20°C cryostat (Leica 1950; Leica Biosystems, Buffalo Grove, IL, USA) and mounted onto Superfrost Plus slides (Fisher Scientific, Hampton, NH, USA). On the first day of staining, sections were fixed for five min using 4% paraformaldehyde at room temperature and rinsed four times with 1XPBS prior to five minutes was in 1XPBS. Sections were placed in 1X Power block for 10 min (Universal Blocking Reagent 10X, Biogenex, Cat#HK085-5 K), repeated rinse and wash with 1XPBS and placed in rabbit anti-GFP primary (1:20,000 in 1%BSA, 1XPBS) overnight at 4°C. The next day, sections were rinsed with 1XPBS then washed three times for three minutes each. Sections were placed into 1.5% H_2_O_2_ for 20 min at RT, washed for four times, three minutes each with 1XPBS and gently dried. Super Picture HRP Polymer Conjugate Rabbit Primary Kit (Invitrogen, Cat#87-9263) was used for the following steps. Each slide had 100 μL of antibody from Rabbit PolyHRP conjugate applied and incubated for 30 min at RT. Washed two times in 1XPBS for three minutes each and one time in 0.1 M Tris (pH8) for three minutes. The DAB step was prepared according to Rabbit PolyHRP directions. Sections were air dried and cover slipped using DPX mounting media. Site checks were then performed with light microscopy.

## Results

### Experiment 1: behavior and immediate early gene activation in virgin pup-sensitized Oxtr FB/FB females

**Figure 1:**
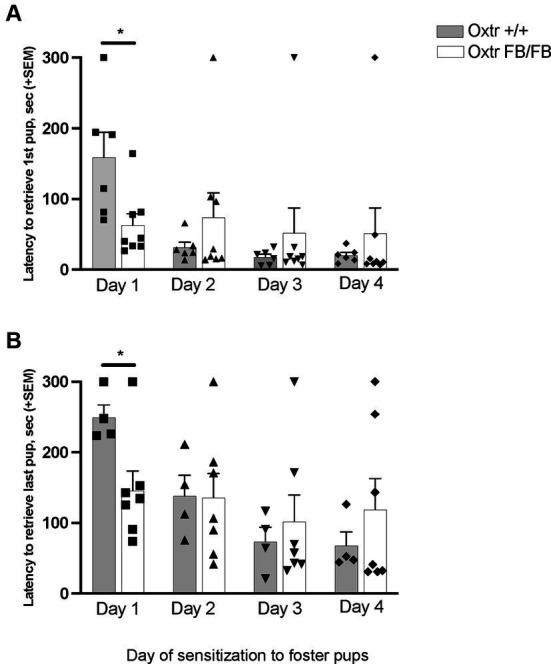
Latency to retrieve **(A)** first pup or **(B)** all pups across four days of pup-sensitization in wildtype (Oxtr +/+) and Oxtr conditional forebrain knockout (Oxtr FB/FB) naïve, virgin females. On Day 1, Oxtr FB/FB females displayed decreases in their latency to retrieve first and all pups. Graphs depict mean + SEM (*p ≤ 0.05).

In pup-sensitized and Oxtr +/+ (*n* = 6) Oxtr FB/FB (*n* = 8) a repeated measures ANOVA revealed a main effect of day of testing for retrieving the first pup (*F*_3,10_ = 5.448, *p* = 0.018) and retrieving all pups (*F*_3,7_ = 19.432, *p* = 0.001) (Figure 1). Additionally, an interaction of day and genotype was found for retrieval of first pup (*F*_3,10_ = 4.203, *p* = 0.036) and retrieval of all pups (*F*_3,7_ = 8.477, *p* = 0.010). *Post hoc* analysis revealed a genotypic difference in the latency to retrieve the first pup (*F*_1,13_ = 7.195, *p* = 0.020) and all pups (*F*_1,9_ = 6.799, *p* = 0.028) on the first day of exposure, with the Oxtr FB/FB females having shorter retrieval latencies compared to Oxtr +/+ females. No genotypic differences in latencies to retrieve first pup or all pups were observed on any of the other days of testing. For the ‘retrieving all pups’ measure only *n* = 4 Oxtr +/+ and *n* = 7 Oxtr FB/FB were included in the analysis as there was pup loss across the days of testing resulting in fewer pups to retrieve.

Pup-sensitized Oxtr +/+ (*n* = 6) and Oxtr FB/FB (*n* = 8) females did not differ in any maternal behaviors observed across testing days (Supplementary Figure S1). No genotypic differences were observed on any of the days. Day 1: duration time on nest (*F*_1,12_ = 2.111, *p* = 0.174), nest building (*F*_1,12_ = 0.148, *p* = 0.708), self-grooming (*F*_1,12_ = 0.021, *p* = 0.887), nonsocial (*F*_1,12_ = 0.083, *p* = 0.778) and pup interactions (*F*_1,12_ = 0.097, *p* = 0.762). Day 2: duration of time on nest (*F*_1,12_ = 0.626, *p* = 0.446), nest building (*F*_1,12_ = 0.182, *p* = 0.678), self-grooming (*F*_1,12_ = 0.023, *p* = 0.882), nonsocial (*F*_1,12_ = 0.012, *p* = 0.915) and pup interactions (*F*_1,12_ = 1.330, *p* = 0.273). Day 3: time on nest (*F*_1,12_ = 0.318, *p* = 0.584), nest building (*F*_1,12_ = 2.757, *p* = 0.125), self-grooming (*F*_1,12_ = 0.003, *p* = 0.959), nonsocial (*F*_1,12_ = 0.720, *p* = 0.414) and pup interactions (*F*_1,12_ = 1.040, *p* = 0.330). Day 4: time on nest (*F*_1,12_ = 1.790, *p* = 0.208), nest building (*F*_1,12_ = 0.913, *p* = 0.360), self-grooming (*F*_1,12_ = 4.486, *p* = 0.058), nonsocial (*F*_1,12_ = 1.142, *p* = 0.308) and pup interactions (*F*_1,12_ = 0.001, *p* = 0.975).

**Figure 2:**
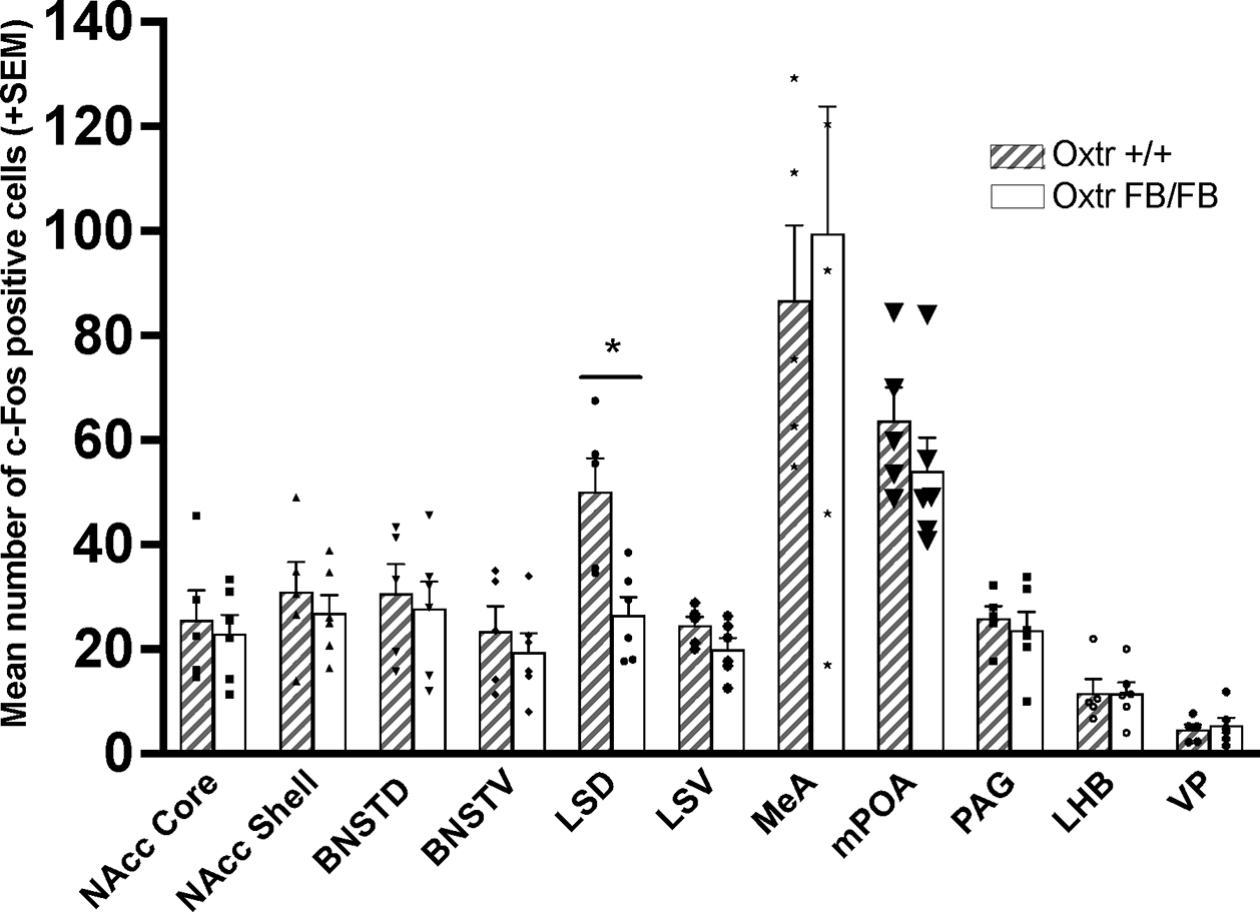
Mean number of c-Fos immunoreactive cells per brain region in wildtype (Oxtr +/+) and Oxtr conditional forebrain knockout (Oxtr FB/FB) females sensitized to pups. On the fourth day of repeated exposure to pups, Oxtr +/+ and Oxtr FB/FB had a significant genotypic difference in c-Fos immunoreactivity within the LSD. No genotypic differences were observed in any of the other measured brain regions. The graph depicts mean + SEM (*p ≤ 0.05). NAcc Core, nucleus accumbens core; NAcc Shell, nucleus accumbens shell; BNSTD, dorsal bed nucleus stria terminalis; BNSTV, ventral bed nucleus stria terminalis; LSD, dorsal lateral septum; LSV, ventral lateral septum; MeA, medial amygdala; mPOA, medial preoptic area; PAG, periaqueductal grey; LHB, lateral habenular nuclei; VP, ventral pallidum.

Oxtr FB/FB sensitized females (*n* = 6) had decreased c-Fos immunoreactivity in the LSD compared to the Oxtr +/+ sensitized females (*n* = 5) (*F*_1,10_ = 11.339, *p* = 0.008) (Figure 2; Supplementary Figure S2); three animals were not included in the c-fos study due to issues with the immunocytochemistry protocol, *n* = 2 Oxtr +/+ and *n* = 1 Oxtr FB/FB. No genotypic differences were observed in the NAcc core (*F*_1,10_ = 0.172, *p* = 0.688), NAcc shell (*F*_1,10_ = 0.393, *p* = 0.546), BNSTD (*F*_1,10_ = 0.143, *p* = 0.714), BNSTV (*F*_1,10_ = 0.453, *p* = 0.518), LSV (*F*_1,10_ = 2.616, *p* = 0.140), MeA (*F*_1,10_ = 0.185, *p* = 0.678), mPOA (*F*_1,10_ = 1.113, *p* = 0.319), PAG (*F*_1,10_ = 0.249, *p* = 0.630), LHB (*F*_1,10_ = 0.001, *p* = 0.973), or the VP (*F*_1,10_ = 0.249, *p* = 0.630).

### Experiment 2: parturition-induced immediate early gene activation in Oxtr −/− and Oxtr FB/FB dams

**Figure 3:**
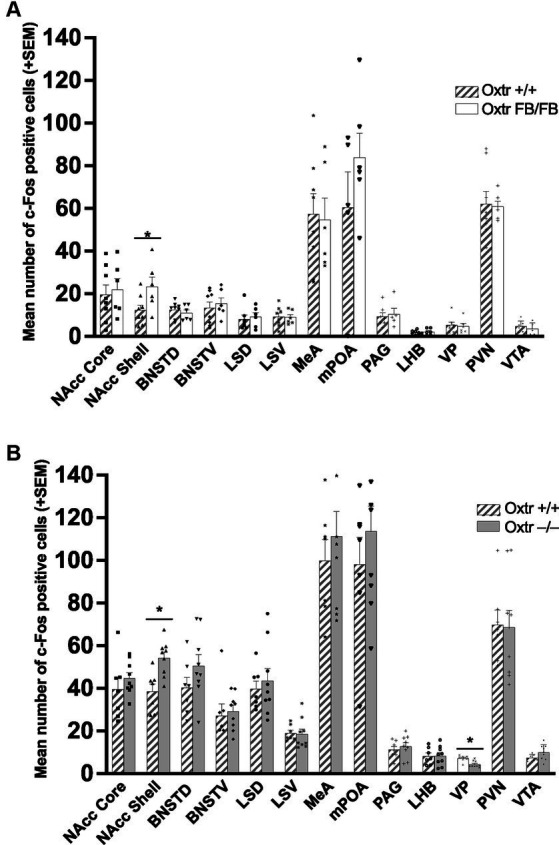
**(A)** Mean number of c-Fos immunoreactive cells per brain region in wildtype (Oxtr +/+) and Oxtr conditional forebrain knockout (Oxtr FB/FB) females following parturition. In one-hour post parturient Oxtr +/+ and Oxtr FB/FB females a significant genotypic difference in c-Fos immunoreactivity was found within the NAcc shell. The Oxtr FB/FB females have an increase in activation in the NAcc shell compared to Oxtr +/+ female. No genotypic differences were observed in any of the other measured brain regions. Graphs depict mean + SEM (*p ≤ 0.05). **(B)** Mean number of c-Fos immunoreactive cells per brain region in wildtype (Oxtr +/+) and Oxtr knockout (Oxtr −/−) females following parturition. In one-hour post-parturient Oxtr +/+ and Oxtr −/− females a significant genotypic difference in c-Fos immunoreactivity was found within the NAcc shell and VP. Oxtr −/− females have an increase in activation in the NAcc shell and decreased activation in the VP compared to Oxtr +/+ female. No genotypic differences were observed in any of the other measured brain regions. Graphs depict mean + SEM (*p ≤ 0.05). NAcc Core, nucleus accumbens core; NAcc Shell, nucleus accumbens shell; BNSTD, dorsal bed nucleus stria terminalis; BNSTV, ventral bed nucleus stria terminalis; LSD, dorsal lateral septum; LSV, ventral lateral septum; MeA, medial amygdala; mPOA, medial preoptic area; PAG, periaqueductal grey; LHB, lateral habenular nuclei; VP, ventral pallidum; PVN, paraventricular nucleus; VTA, ventral tegmental area.

In the one-hour post-parturient Oxtr +/+ (*n* = 8) and Oxtr FB/FB (*n* = 6) dams there was a significant genotypic difference in c-Fos immunoreactivity within the NAcc shell (*F*_1,13_ = 5.192, *p* = 0.042), with an increase in activation in Oxtr FB/FB dams compared to Oxtr +/+ dams (Figure 3–top panel; Supplementary Figure S3). No genotypic differences in c-Fos activation were observed in any of the other measured brain regions: BNSTD (*F*_1,13_ = 0.594, *p* = 0.456), BNSTV (*F*_1,13_ = 0.257, *p* = 0.621), LHB (*F*_1,13_ = 0.330,*p* = 0.576), LSD (*F*_1,12_ = 0.163, *p* = 0.694), LSV (*F*_1,13_ = 0.021, *p* = 0.888), NAcc core (*F*_1,13_ = 0.130, *p* = 0.725), MeA (*F*_1,13_ = 0.036, *p* = 0.852), mPOA (*F*_1,10_ = 1.421, *p* = 0.264), PAG (*F*_1,11_ = 0.106, *p* = 0.751), PVN (*F*_1,13_ = 0.027, *p* = 0.872), VP (*F*_1,13_ = 0.608, *p* = 0.451) or VTA (*F*_1,13_ = 0.597, *p* = 0.457).

In the one-hour post-parturient Oxtr +/+ (*n* = 7) and Oxtr −/− (*n* = 9) dams there was a significant genotypic difference in c-Fos immunoreactivity within the NAcc shell (*F*_1,15_ = 12.756, *p* = 0.03) with an increase in activation in Oxtr −/− dams compared to Oxtr +/+ dams (Figure 3–bottom panel; Supplementary Figure S4) and the VP (*F*_1,15_ = 12.938, *p* = 0.003) with an increase in activation in Oxtr +/+ dams. No genotypic differences in c-Fos expression were observed in any of the other measured brain regions: BNSTD (*F*_1,15_ = 1.990, *p* = 0.180), BNSTV (*F*_1,15_ = 0.133, *p* = 0.721), LHB (*F*_1,15_ = 0.004, *p* = 0.950), LSD (*F*_1,15_ = 0.284, *p* = 0.603), LSV (*F*_1,15_ = 0.032, *p* = 0.860), MeA (*F*_1,15_ = 0.512, *p* = 0.486), mPOA (*F*_1,15_ = 0.781, *p* = 0.392), NAcc core (*F*_1,15_ = 0.856, *p* = 0.371), PAG (*F*_1,15_ = 0.419, *p* = 0.528), PVN (*F*_1,15_ = 0.012, *p* = 0.916), or VTA (*F*_1,15_ = 3.354, *p* = 0.088).

### Experiment 3: effects of targeted genetic knockdown of the Oxtr on maternal behavior

#### Experiment 3a: nucleus accumbens

**Figure 4:**
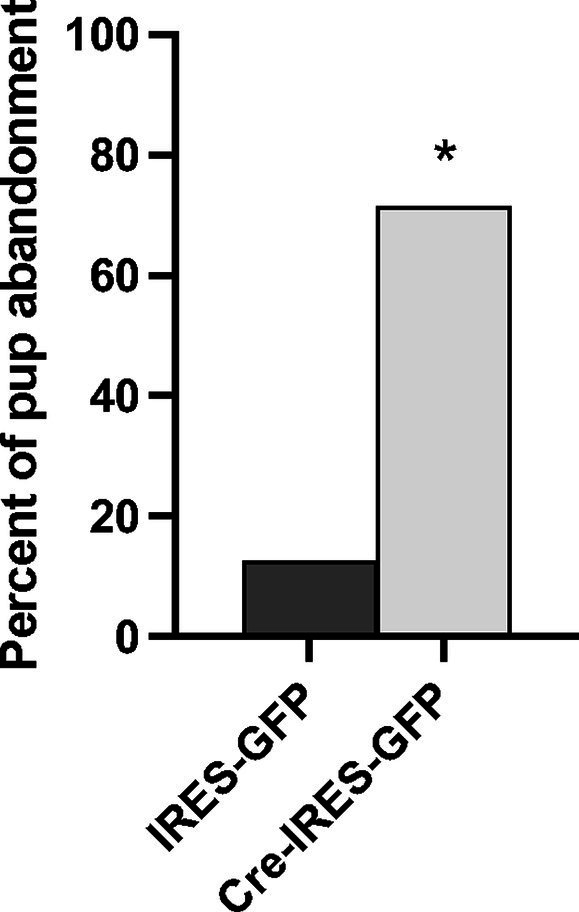
Percent of litters abandoned by Oxtr flox/flox dams that received either control (IRES-GFP) or Cre recombinase (Cre-IRES-GFP) targeted injections in the nucleus accumbens shell. Females injected with Cre-IRES-GFP displayed increased pup abandonment by postnatal day 2 compared to dams injected with IRES-GFP (*p ≤ 0.05).

In total, *n* = 8 IRES-GFP and *n* = 7 Cre-IRES-GFP female littermates were evaluated in Experiment 3a. Site checks verified that the viral injections were all within the NAcc shell, though a few were prior to or immediately after the targeted bregma position at +1.58 anterior posterior (Supplementary Figure S5). One Cre-IRES-GFP animal was removed from the experiment due to disturbances in the animal facility during testing. No notable differences in general health of the dam or pups on PND0 were observed between treatment groups. All pups were found alive on PND0, except for one Cre-IRES-GFP injected dam. Since most litter losses occurred by PND1, pup weights were only compared on PND0 and no treatment specific differences were observed (*F*_1,12_ = 1.114, *p* = 0.287). A Fisher’s exact test identified a significant genotypic difference in pup abandonment as a function of treatment (*p* = 0.023) (Figure 4). 71% of females injected with Cre-IRES-GFP abandoned their litters by PND2 (5 out of 7), with all pups cannibalized or found dead in their cage. In contrast, only 12.5% of dams injected with IRES-GFP abandoned their litters by PND2 (1 out of 8). This pup abandonment extended to second litters with 40% (2 out of 5) of Cre-IRES-GFP dams abandoning their litters compared to 0% (0 out of 1) of IRES-GFP dams. Regardless of litter survival, all dams were tested for anxiety-like behavior in the elevated plus maze and the open field, and for depression-like behavior in the forced swim test (Supplementary Figure S6). No treatment-specific differences were observed in the percent time spent on the open arms of elevated plus (*F*_1,14_ = 0.126, *p* = 0.728), the percent time in the inner arena of open field (*F*_1,14_ = 0.337, *p* = 0.572), or percent time spent swimming in the forced swim test (*F*_1,14_ = 0.249, *p* = 0.626). Due to the high percentage of pup abandonment in Cre-IRES-GFP dams, no maternal behaviors were analyzed.

#### Experiment 3b: posterior paraventricular thalamus

In total, *n* = 5 Cre-IRES-GFP and *n* = 6 IRES-GFP female littermates were evaluated in Experiment 3b. Site checks verified that the viral injections were all within the pPVT, though a few were slightly to the right of the targeted bregma position at 0.0 on the medial lateral axis (Supplementary Figure S7). All pups, except for those from two IRES-GFP dams, were found alive on PND0. A Fisher’s exact test determined that there was no significant genotypic difference in pup abandonment as a function of treatment (two-tailed *p* = 0.545). Specifically, 20% of females injected with Cre-IRES-GFP abandoned their litters by PND1, with all pups cannibalized or found dead in their cage, and 50% of dams injected with IRES-GFP abandoned their litters by PND1. For dams that did not abandon their pups, maternal behaviors were tested, and treatment specific differences were observed only in time spent self-grooming (*F*_1,4_ = 11.628, *p* = 0.027). No treatment specific differences were found in latency to retrieve the first pup (*F*_1,4_ = 4.287, *p* = 0.107), latency to all pups (*F*_1,4_ = 4.576, *p* = 0.099), time spent off the nest (*F*_1,4_ = 0.184, *p* = 0.690), time spent licking/sniffing (*F*_1,4_ = 0.210, *p* = 0.670), and time spent rearing (*F*_1,4_ = 0.124, *p* = 0.743). Maternal behavior data can be found in Supplementary Table S1. There were no treatment-specific differences in the percent time spent on the open arms of elevated plus maze (*F*_1,8_ = 0.325, *p* = 0.288). However, there were treatment-specific differences in the percent time spent in the inner portion of the open field (*F*_1,8_ = 0.901, *p* = 0.044) and in the percent time spent swimming in the forced swim test (*F*_1,8_ = 4.074, *p* = 0.004) with Cre-IRES-GFP females having reductions in both as compared to IRES-GFP females, which would suggested increased anxiety-like and depression-like behavior. Elevated plus, open field, and forced swim data can be found in Supplementary Table S2.

## Discussion

The work presented here suggests that pup-sensitized and post-parturient females differ neurochemically from one another. Most importantly, a neuroanatomical substrate where Oxt signaling through Oxtr mediates the onset of maternal behavior in post-parturient females was identified. Specifically, increased c-Fos immunoreactivity in one-hour post-parturient Oxtr −/− and Oxtr FB/FB dams established that the NAcc shell was important. Though, whether or not it was Oxtr signaling that was responsible for the increase in c-Fos activation, or some downstream effect, was unknown. However, a targeted genetic disruption of Oxtr expression within NAcc shell resulted in a pup abandonment phenotype similar to that observed in Oxtr −/− (Rich et al., 2014) and Oxtr FB/FB dams (Macbeth et al., 2010), thus implicating Oxtr signaling in the NAcc shell as being important for the onset of maternal behavior. Specifically, 71% of dams injected with Cre-IRES-GFP abandoned their pups. Further, the effects of Oxtr knockdown on pup abandonment were anatomically specific to the NAcc, as knockdown of the Oxtr within the pPVT resulted in no significant change in pup abandonment as compared to controls.

In virgin pup-sensitized Oxtr FB/FB females there were no observed differences in c-Fos immunoreactivity in the NAcc shell, as was observed in post-parturient Oxtr −/− and Oxtr FB/FB dams. Rather, we found a difference in the LSD, with Oxtr FB/FB having less activation than controls. Based on known differences between pup-sensitized females and post-parturient dams, this finding was not completely surprising (Matsushita et al., 2015). The LS helps to regulate emotional processes and stress responses via dense interconnections with several limbic, diencephalic, and midbrain regions and has connectivity to the MBNN at the BNST, NAcc, PAG, and VTA (Sheehan et al., 2004). Further, in dams, decreases in social fear are associated with reduced activation of the LS but increases in Oxt fiber density from the SON to the LS and in turn increases in Oxt release (Menon et al., 2018). The Oxtr is also expressed in the LS and there is evidence that Oxt signaling in the LS can enhance social memory, which is important for maternal behavior (Loup et al., 1991; Popik et al., 1992; Barberis and Tribollet, 1996). We postulate that activation of the Oxtr in the LSD is increased in pup-sensitized Oxtr +/+ females, relative to pup-sensitized Oxtr FB/FB females, due to repeated exposure to pups. This idea is supported by a study in which pup-sensitized females, but not females that were exposed to pups one at a time, show increases in neuronal activity in the mPOA and Oxt system (Okabe et al., 2017). It is also consistent with data demonstrating that the LSD, specifically, is important to the control reward-driven behaviors (Puska et al., 2024). However, the behavioral impact of this genotyptic difference in neuronal activity in the LSD is unknown, as the core aspects of maternal behavior, e.g., pup retievals, nest building, and pup interactions, were not affected on the day of tissue collection (Day 4).

The only behavioral differences between virgin Oxtr FB/FB females and control females were found on Day 1 of testing, with virgin Oxtr FB/FB females having decreased latencies in the retrieval of the first pup and all pups compared to virgin Oxtr +/+ females. As pup avoidance is not atypical of inexperienced females, with repeated exposure increasing care giving behaviors (Stolzenberg and Champagne, 2016), the reduced latencies to retrieve pups on Day 1 in virgin Oxtr FB/FB seems unusual as an absence of Oxtr signaling tends to be associated with higher levels of stress and anxiety. However, Oxt signaling does not always reduce anxiety and fear. The emotional state of the animal is important as is where in the brain the Oxtr signaling is occurring. For instance, Oxt signaling in the central amygdala has bidirectional effects, sometimes enhancing and sometimes potentiating fear (van den Burg and Hegoburu, 2020). Thus, it is possible that the loss of Oxtr signaling in the forebrain reduced social fear allowing for better maternal responsiveness in the form of pup retrievals even with inexperience. To identify possible the brain regions involved c-Fos immunoreactivity across the days of sensitization could be quantified in Oxtr +/+ and Oxtr FB/FB females.

Beyond day 1, there were no genotypic differences in maternal behaviors following pup-sensitization, which is consistent with the previously reported normal maternal care in post-parturient Oxtr FB/FB dams (Rich et al., 2014). Certainly, pup-sensitized females do differ from non-pup-sensitized females. For instance, while laboratory mice display spontaneous maternal behavior characterized by pup retrievals and the adoption of a nursing posture within minutes of exposure (Olazábal et al., 2013a), only pup-sensitized virgins will retrieve pups from a novel environment back to their home cages (Stolzenberg and Numan, 2011). Our observed differences in c-Fos activation in pup-sensitized, as compared to post-parturient females, suggests that to understand the maternal brain it might be preferable to focus on pre- and post-parturient females rather than pup-sensitized females. The findings of Experiment 2 provide evidence in support of our *hypothesis*, as they suggest that the pup abandonment phenotype previously observed in Oxtr −/− and Oxtr FB/FB females (Macbeth et al., 2010; Rich et al., 2014) is likely due to a failure of the brain to “switch” to a more maternal state. The data from Experiment 3 suggest that this “switch” is likely located in the NAcc shell.

Genetic disruption of Oxtr expression in the NAcc shell, but not the pPVT, resulted in a robust pup abandonment phenotype, which suggests that Oxtr expression in the NAcc shell is a critical for the onset of maternal behavior in post-parturient females. Interestingly, there are known species differences in Oxtr expression within the NAcc, with Oxtr expression being generally higher in species that are more responsive to their young (Olazábal et al., 2013b). In prairie voles, for instance, Oxtr expression in the NAcc has been established as being important for the neural regulation of alloparental care, i.e., parental care toward non-descendant young, and pair bond formation (Insel and Shapiro, 1992; Young and Wang, 2004; Olazabal and Morrell, 2005; Olazabal and Young, 2006; Keebaugh and Young, 2011). Though, the work described here is the first to functionally link Oxtr expression in the NAcc to maternal care in post-parturient mice. It is important to note that genetic disruption of the Oxtr within the pPVT, while not impacting the onset of maternal behavior, did result in decreased self-grooming and increased anxiety- and depression-like behaviors. Given the involvement of the pPVT in fear and stress, as well as in Oxt-regulated crouching behavior (Watarai et al., 2020; Kooiker et al., 2021), it seems that more work will be need to be performed to better understand these data and the possible implications for a maternal brain.

The NAcc is part of the MBNN, which is important for maternal motivation and memory. Importantly, Oxtr are expressed in the NAcc, and appear to form heterocomplxes with the dopamine (DA) D2 receptor protomers allowing for responsiveness to the co-release of Oxt and DA (Romero-Fernandez et al., 2013; Borroto-Escuela et al., 2022). In hormonally primed females, DA is released into the NAcc following activation of the VTA, via projections from the mPOA/vBNST. The NAcc also receives afferent projections from the BLA/PFC and DA is known to dampen the response to BLA/PFC input. Thus, when DA is present there is a dampening of NAcc output. The consequence of this is that the VP is released from GABAergic inhibition, this disinhibition is thought to allow the VP to be responsive to pup stimuli—which is permissive for appetitive maternal behaviors (Numan et al., 2005). In this study while we were not evaluating the dampening of NAcc output, we did observe increased immediate early gene activation in the NAcc of both Oxtr FB/FB or Oxtr −/− dams. Thus, we speculate that genetic disruption of Oxtr signaling resulted in the GABAergic inhibition of the VP being maintained, which would in turn inhibited the onset of maternal behavior.

## Conclusion

The presented here shed light on the importance of the Oxt system and specifically the Oxtr in virgin pup-sensitized and post-parturient maternal behavior. Differential neuronal activation was observed depending on the presence, or absence (sensitization), of hormonal changes during parturition. Based on our findings from Experiment 1, we hypothesize that the increased maternal responsiveness, as measured by reduced latencies to retrive pups in Oxtr FB/FB virgin pup-sensitized females on Day 1 of testing, may be due to decreased social fear and increased maternal responsiveness. Though, more work will need to be done to identify possible neural substrates. In Experiment 2, the NAcc shell was identified as a neuroanatomical region where Oxtr signaling may play a role in the onset of maternal care in post-parturient females. This was confirmed in Experiment 3 when a targeted knockdown of Oxtr in the NAcc shell resulted in a robust pup abandonment phenotype, similar to what has previously been observed in Oxtr FB/FB and Oxtr −/− dams (Rich et al., 2014). While addition work is needed to determine how Oxtr signaling in the NAcc is modulated, there is evidence that PVN Oxt neurons respond to pup vocalizations and can control Oxt release (Valtcheva et al., 2023). So, perhaps in the right hormonal state the salience of the pup vocalizations results in increased Oxt signaling via the Oxtr in the NAcc which reinforces the DA signal and enhances the disinhibition of the VP ultimately lowering the threshold for the onset of maternal behavior. This enhancing of a signal would be consistent with the modulatory effects of the Oxt system. Importantly, given the high degree of conservation in the Oxt system across species, it is likely that its role in the onset of maternal behavior may extend to species throughout the class Mammalia.

## Data availability statement

The raw data supporting the conclusions of this article will be made available by the authors, without undue reservation.

## Ethics statement

The animal study was approved by Kent State University Institutional Animal Care and Use Committee. The study was conducted in accordance with the local legislation and institutional requirements.

## Author contributions

SW: Conceptualization, Formal analysis, Investigation, Methodology, Writing – original draft, Writing – review & editing. AH: Formal analysis, Investigation, Methodology, Writing – original draft, Writing – review & editing. HC: Conceptualization, Formal analysis, Funding acquisition, Methodology, Project administration, Resources, Supervision, Writing – original draft, Writing – review & editing.
