# Supplementary figures and images for: Oxytocin receptors in the nucleus accumbens shell are necessary for the onset of maternal behavior
Source: Front Neurosci. 2024 Jul 2;18:1356448. doi: 10.3389/fnins.2024.1356448 (PMC11250266; doi:10.3389/fnins.2024.1356448)

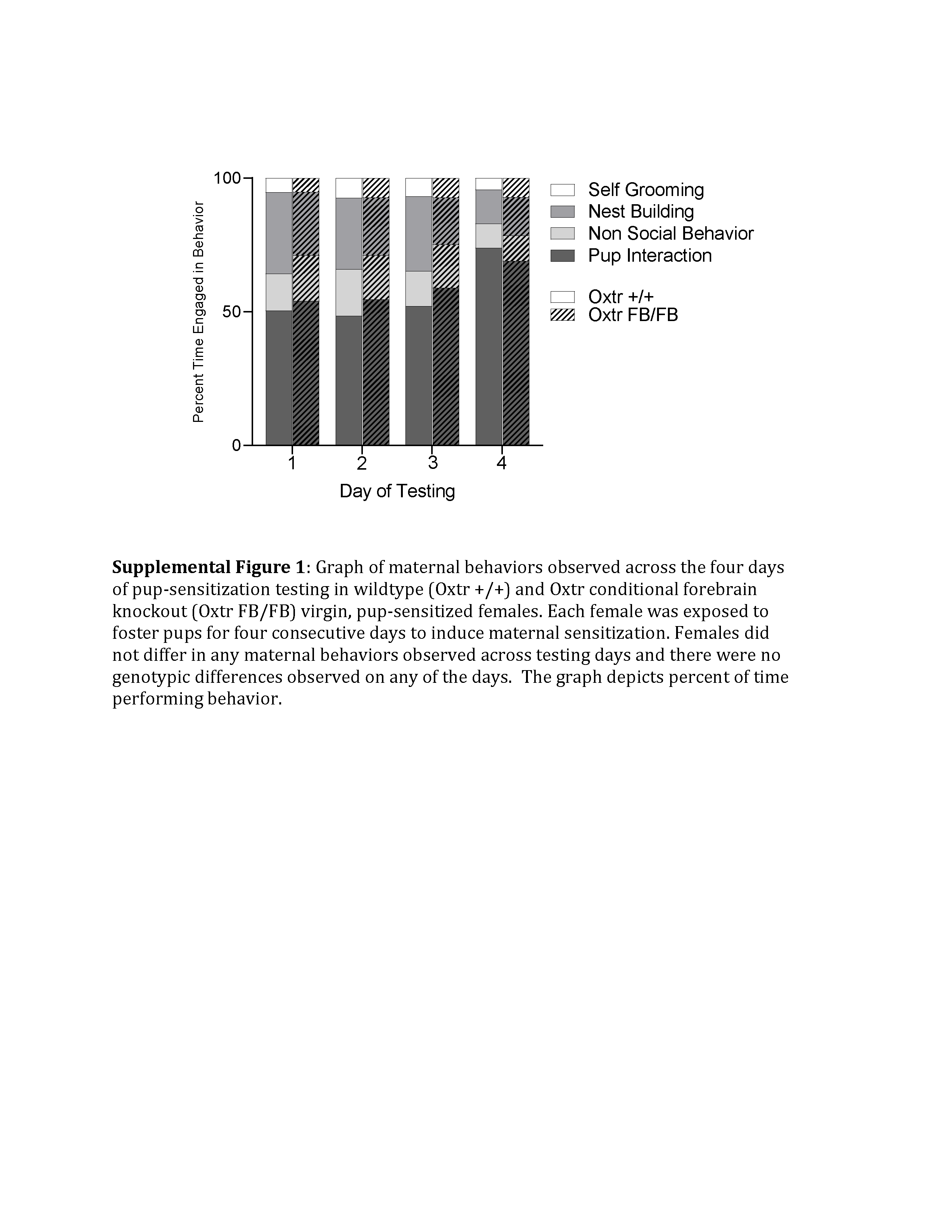

Supplement: Supplementary file 1 [file Image_1.tiff]

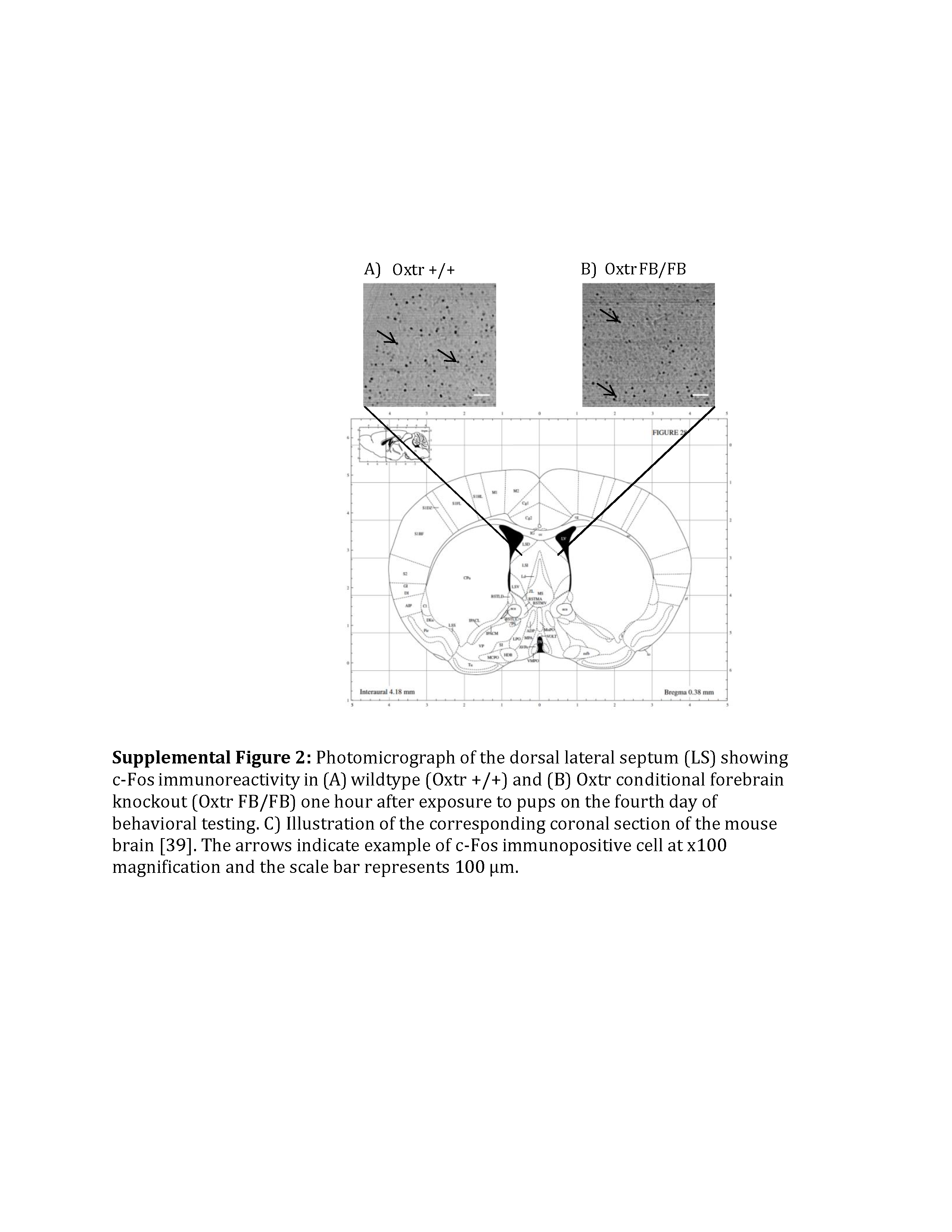

Supplement: Supplementary file 2 [file Image_2.tiff]

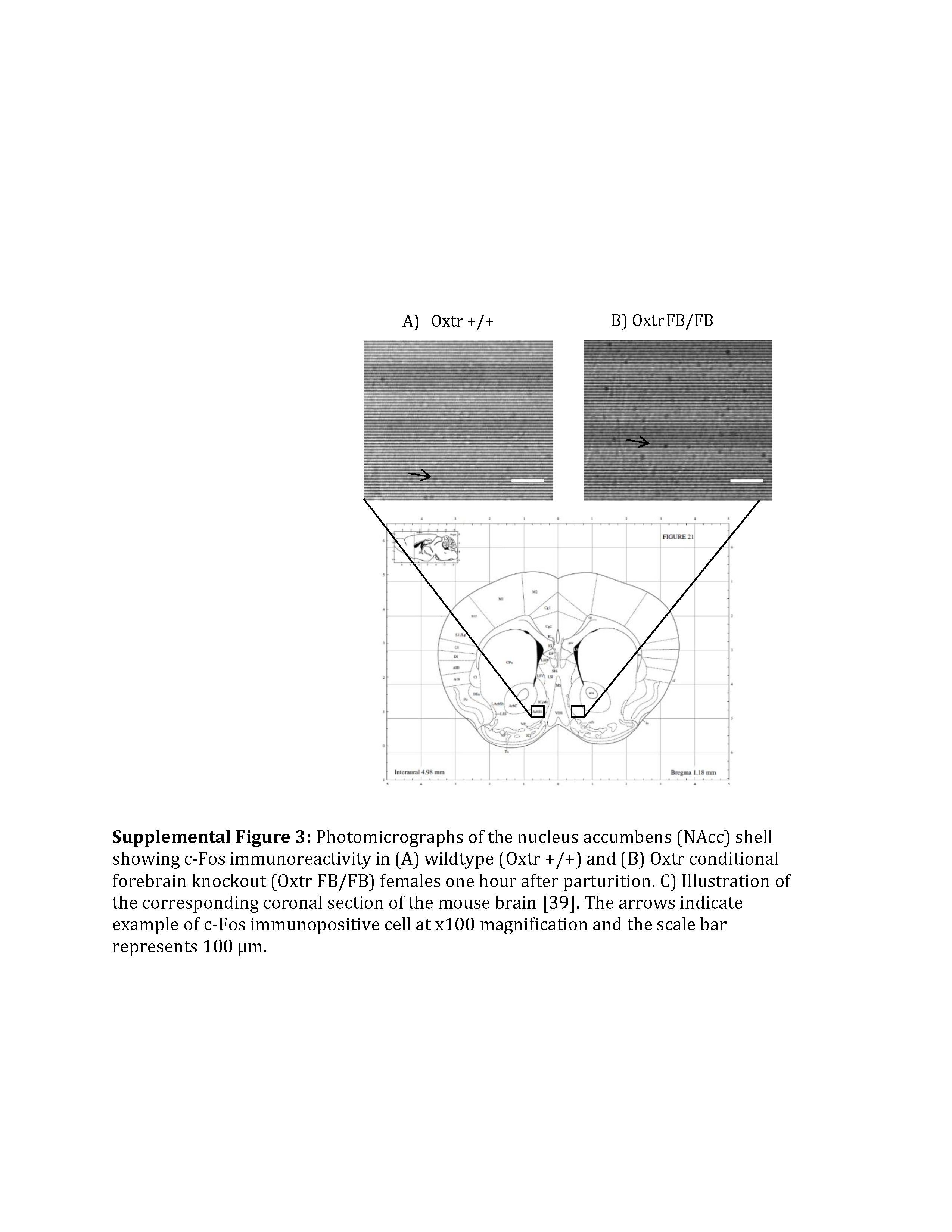

Supplement: Supplementary file 3 [file Image_3.tiff]

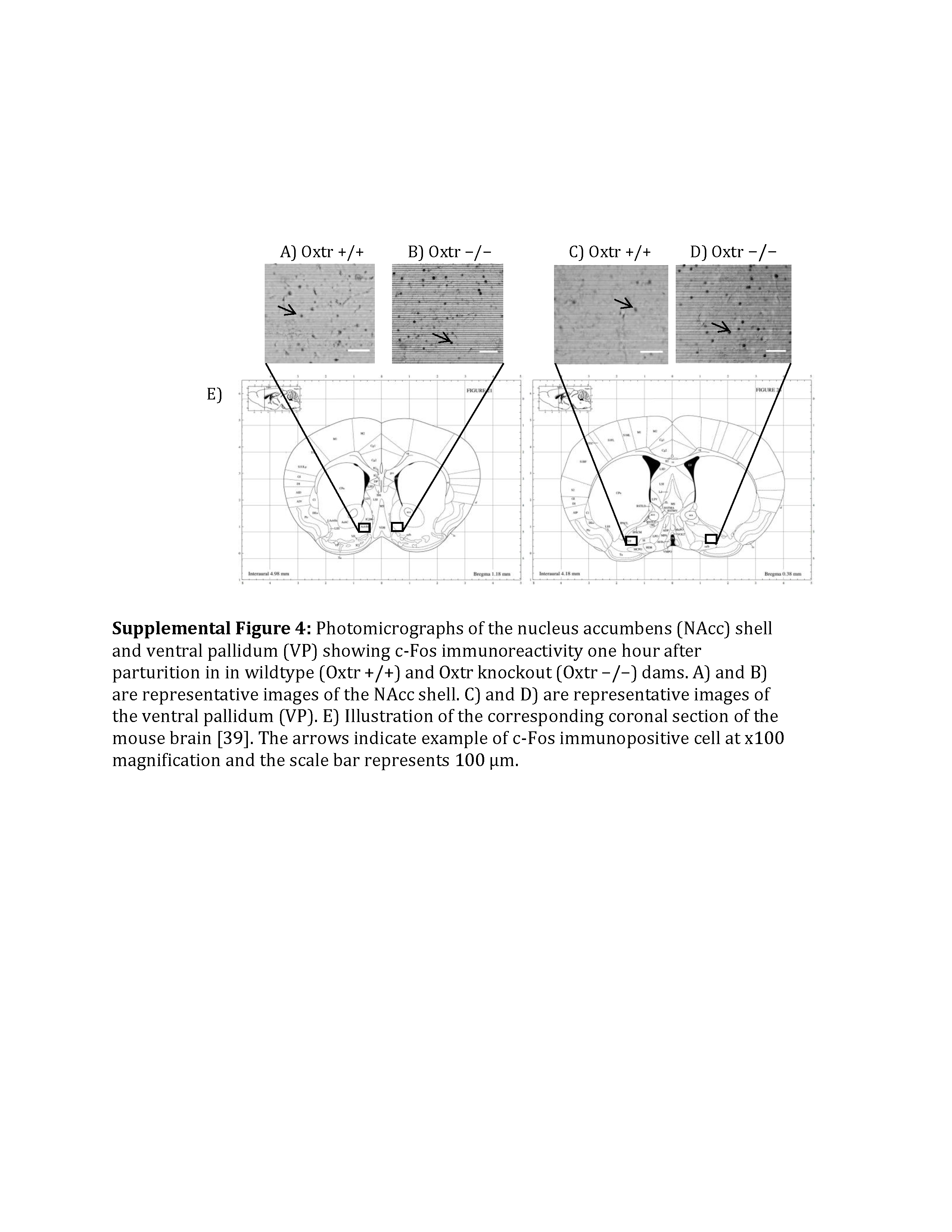

Supplement: Supplementary file 4 [file Image_4.tiff]

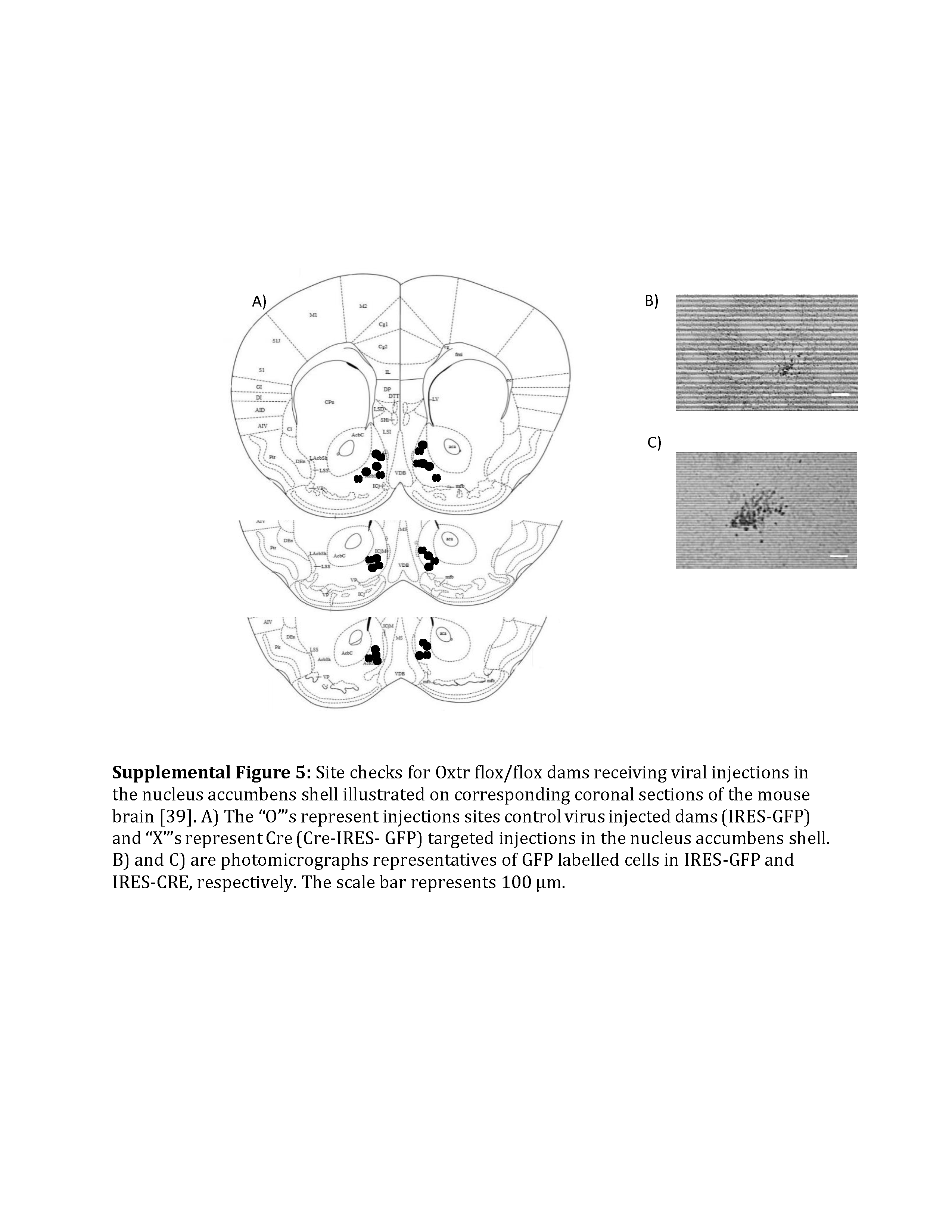

Supplement: Supplementary file 5 [file Image_5.tiff]

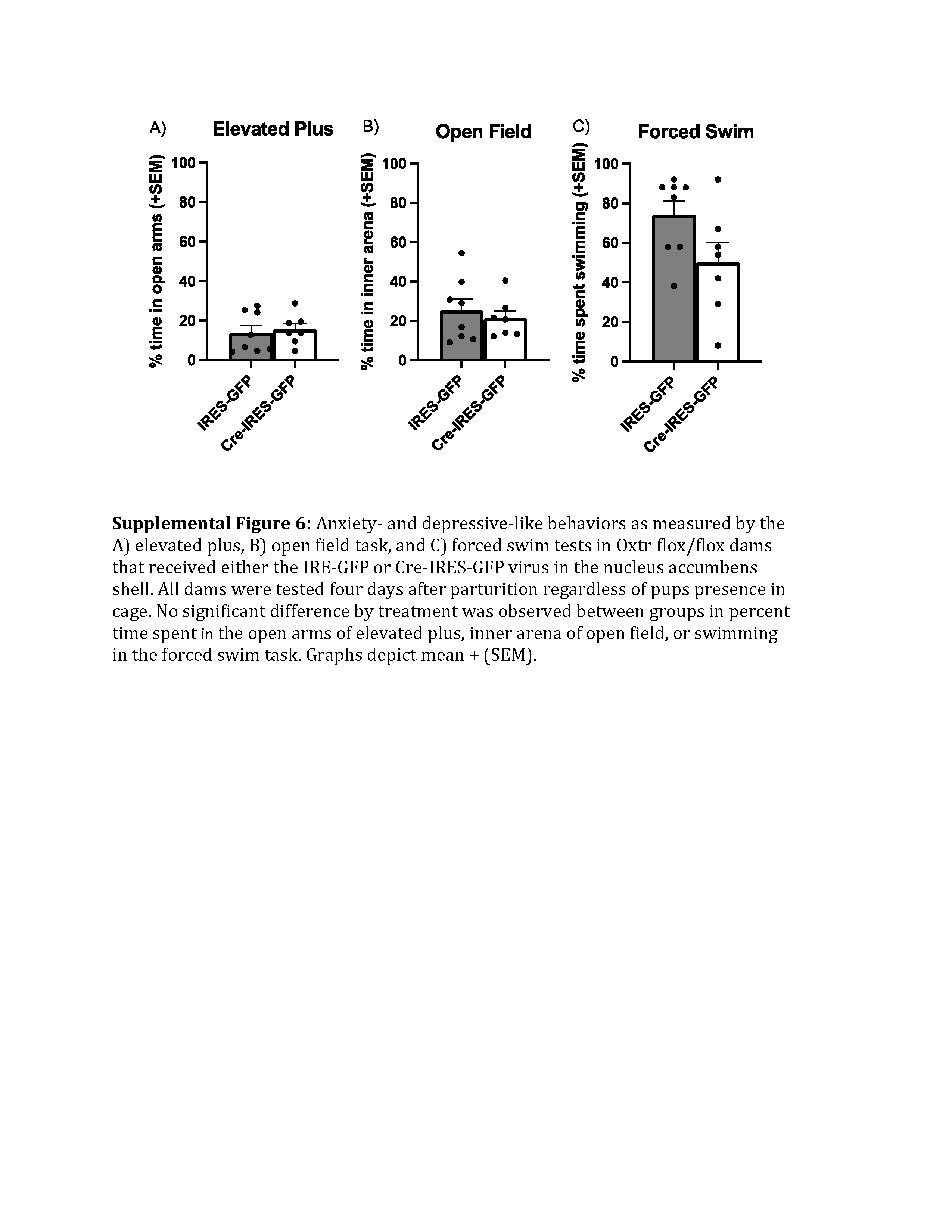

Supplement: Supplementary file 6 [file Image_6.tiff]

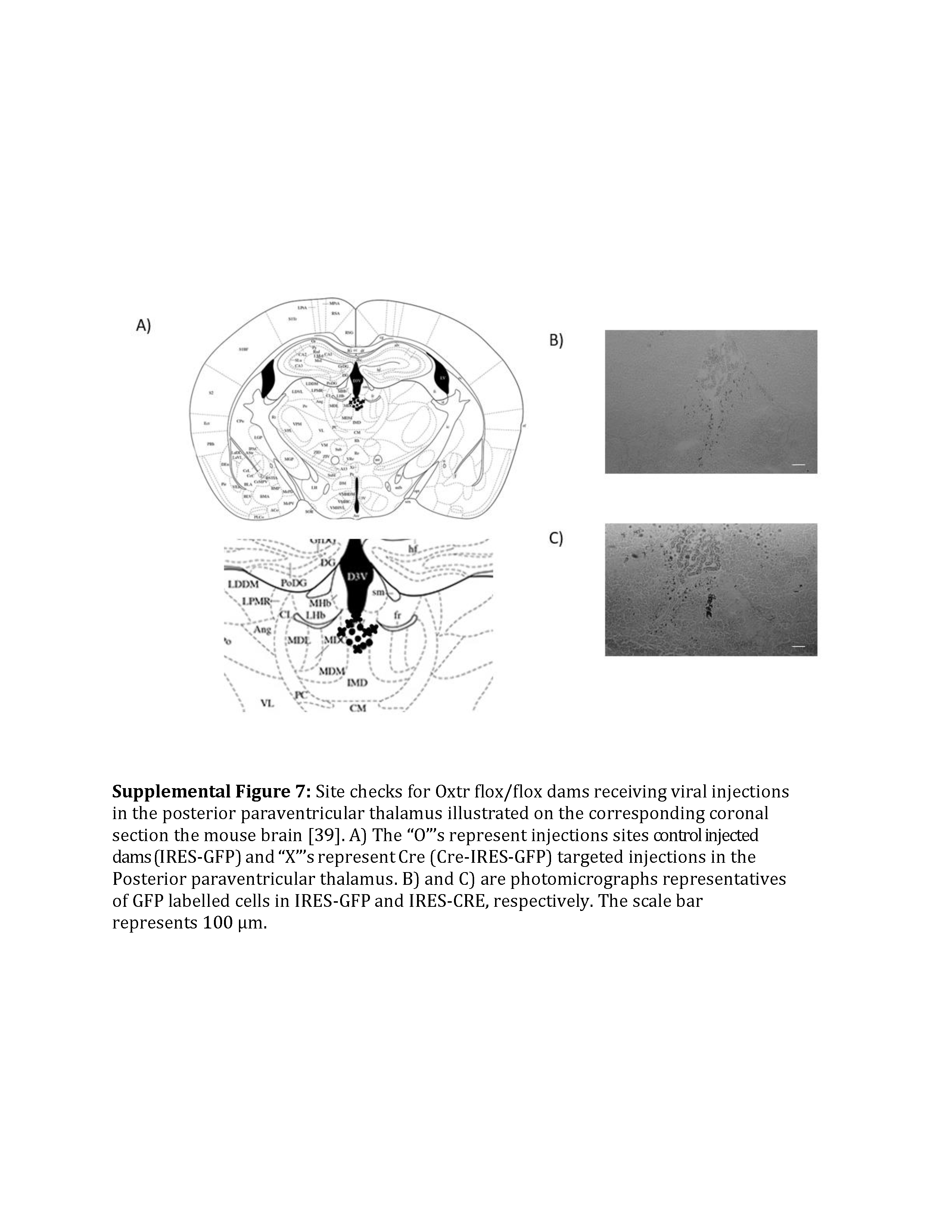

Supplement: Supplementary file 7 [file Image_7.tiff]
